# Advances in molecular basis of response to immunotherapy for penile cancer: better screening of responders

**DOI:** 10.3389/fonc.2024.1394260

**Published:** 2024-07-17

**Authors:** Da-Ming Xu, Ling-Xiao Chen, Xiao-Yu Zhuang, Hui Han, Miao Mo

**Affiliations:** ^1^ State Key Laboratory of Oncology in South China, Guangdong Provincial Clinical Research Center for Cancer, Sun Yat-sen University Cancer Center, Guangzhou, China; ^2^ Department of Urology, Sun Yat-sen University Cancer Center, Guangzhou, China; ^3^ Department of Urology, Xiangya Hospital, Central South University, Changsha, China; ^4^ Department of Anesthesiology, Second Affiliated Hospital of Shantou University Medical College, Shantou, China

**Keywords:** penile cancer, immunotherapy, biomarker, sensitivity, resistance

## Abstract

Penile cancer is a rare malignant tumor of the male urinary system. The treatment benefit of standard first-line chemotherapy is not ideal for patients with locally advanced or metastatic lymph nodes. Immunotherapy has brought new treatment strategies and opportunities for patients with penile cancer. At present, clinical studies on immunotherapy for penile cancer have been reported, and the results show that it is effective but not conclusive. With the development of immunotherapy and the progress of molecular research technology, we can better screen the immunotherapy response population and explore new combination treatment regimens to evaluate the best combination regimen and obtain the optimal treatment options, which is also an important research direction for the immunotherapy of penile cancer in the future.

## 1 Introduction

Penile cancer is a rare malignant tumor, primarily consisting of squamous cell carcinoma (1). The incidence rate in Western countries ranges from 0.5 to 0.94 per 100,000 males (2). However, certain regions in Africa, South America, and Asia exhibit higher incidence rates, accounting for approximately 1% of male malignancies (2). Lymph node metastasis is a crucial turning point in the prognosis of penile cancer (3). The 5-year tumor-specific survival rate decreases from 95% to 35% when transitioning from lymph node-negative (N0) to lymph node-positive (N3) disease (4). The management of advanced penile cancer poses significant challenges, often employing neoadjuvant therapy followed by consolidative surgery. The TIP regimen (paclitaxel, ifosfamide, and cisplatin) is the standard neoadjuvant treatment recommended in guidelines. However, its clinical efficacy remains unsatisfactory, with an overall survival rate of only 17.1 months, objective response rate of 50.0%, and pathological complete response rate of 10% (5). For advanced patients, there is a need to develop more effective neoadjuvant treatment strategies. Recent studies on immuno-monotherapy or immunotherapy combined have demonstrated its potential benefit in advanced penile cancer patients, albeit with varying responses among individuals (6, 7). Therefore, to better identify responders to immunotherapy in clinical practice, our study provided a comprehensive review of the research progress on the molecular basis of immunotherapy sensitivity and resistance in advanced penile cancer.

## 2 Overview of immunotherapy for penile cancer

In 2019, a 79 years old male diagnosed with metastatic penile cancer who had failed chemotherapy and radiation therapy underwent approximately 2 years of treatment with atezolizumab and achieved nearly complete remission of the cancer (8). Since then, the value of immunotherapy in the treatment of advanced penile cancer has become increasingly prominent (9). However, some discouraging reports on individual cases of immunotherapy failure have prompted a reevaluation of its limitations and scope of application (10). These treatments demonstrate the complexity of immunotherapy in personalized therapy. It is recommended that all relevant immunotherapies be explored from a broader perspective, especially given their differences in the molecular profile of target expression. This approach contributes to a deeper understanding of how various immunotherapies utilize different molecular markers to modulate a patient’s immune response, leading to more effective treatment strategies.

Currently, there are several promising directions for the future of immunotherapy in penile cancer (11). These include immune checkpoint inhibitors (ICIs) blocking PD-1, PD-L1, or CTLA-4 targets, therapeutic vaccines for human papillomavirus (HPV) to induce anti-tumor immune responses in HPV-positive tumors, adoptive immunotherapy containing chimeric antigen receptor (CAR-T) therapy, T cell receptor (TCR) therapy, and tumor-infiltrating lymphocyte (TIL) therapy. The main difference between immune checkpoint inhibitors and other immunotherapy strategies lies in their approach and target. Immune checkpoint inhibitors specifically target the immune escape mechanisms utilized by tumors. These mechanisms hinder the immune system’s ability to attack cancer cells, and checkpoint inhibitors work by releasing these inhibitions, thereby enhancing the immune response to eliminate the tumor. On the other hand, other immunotherapy strategies, such as tumor vaccines and gene therapy, primarily aim to stimulate or modify the patient’s own immune response against cancer cells. Unlike immune checkpoint inhibitors, these strategies do not specifically target certain immune checkpoints but rather regulate or enhance the immune response through different mechanisms to achieve therapeutic effects. Additionally, combination immunotherapies are also being explored. Furthermore, an increasing number of molecular research techniques are being applied to map the genetic and protein expression profiles of penile cancer, including whole exome sequencing, whole genome methylation profiling, DNA copy number profiling, single-cell sequencing of penile cancer (12). Therefore, in the context of exploring immunotherapy, it is important to delineate the potential mechanisms of immunotherapy response events at the molecular level in penile cancer. This will help in accurately assessing the potential efficacy of immunotherapy in advanced penile cancer and identifying the responsive population, providing a reference for future prospective studies on neoadjuvant immunotherapy.

## 3 Molecular basis of immunotherapy response in penile cancer

### 3.1 Tumor mutational burden

TMB is recognized as an important marker of therapeutic sensitivity to ICIs, which refers to the overall number of mutant copies present in the cancer sample (13). Higher TMB is strongly connected with more effective immune therapy response (13). The mechanism behind this is that when immune checkpoint proteins such as PD-1, PD-L1, and CTLA-4 are blocked, the immune system is reactivated and recognizes cancer cells (14). If cancer cells have more immunogenic new antigens, the antigen presentation effect will be more enhanced, and immunogenic new antigens are generally generated by mutations. Therefore, the more mutations there are (higher TMB), the more easily cancer cells can be recognized and eliminated by the immune system (15). Studies have indicated that penile cancer with high TMB responds better to ICIs than those with low TMB (16). However, in penile cancer, there is a complete mismatch repair system and microsatellite stability that maintain a low TMB status (17, 18). The potential association between HPV status and mutational burden in cancers is an intriguing area.

HPV-associated cancers generally exhibit a lower mutational burden compared to non-HPV-associated cancers. We suspect that this is primarily attributed to the role of HPV oncoproteins (E6 and E7) in altering cellular pathways without inducing widespread genomic instability. Interestingly, not all penile cancer patients have high TMB, and the TMB level of metastatic penile cancer is generally higher than that of non-metastatic penile cancer (19). In addition, there was no statistically significant difference between HPV (+) and HPV (-) penile cancers in terms of TMB levels (20). This suggests that patients with metastatic penile cancer may have a better response to ICIs. Moreover, as for the relationship between penile cancer and TMB (tumor mutation load) and MSI (microsatellite instability), there is indeed a lack of sufficient research support to make a clear association between them. Much of the research on mismatch repair system and microsatellite stability has focused on more common cancer types, such as colorectal and lung cancers. Penile cancer is a rare type of cancer, and its genetic characteristics and pathogenesis may differ from other cancers, so further research is needed to explore its potential relationship with mismatch repair system and microsatellite stability. Therefore, the use of ICIs in patients with metastatic penile cancer is undoubtedly a promising and worthy direction for further exploration.

### 3.2 Expression of immune checkpoints

Immune checkpoints such as PD-1, PD-L1, and CTLA-4 are believed to assist tumor cells in evading the immune destruction by CD8+ cytotoxic T cells (21). The use of ICIs is currently a first-line treatment for various metastatic tumors (22). However, only about half of primary penile cancer patients express PD-L1 in their pathological tissues (23, 24), and PD-L1 expression is associated with poor prognosis and lymph node metastasis in penile cancer (25, 26). The prognostic significance of PD-1 target and CTLA-4 target expression in penile cancer has not been confirmed by research. Prospective studies on ICIs in penile cancer have mainly focused on advanced cancer patients with metastasis (27). Additionally, due to the preliminary recognition of the safety and effectiveness of combined treatment strategies involving ICIs with chemotherapy, targeted therapy, or radiotherapy in the treatment of other advanced malignant cancers (28, 29), combination therapy has become an important prospect in the therapeutic regimen of patients with advanced penile cancer. However, the response is limited to only a portion of patients, which is also a core reason for the failure of immunotherapy in other malignant tumors at present (30). Can the expression of immune checkpoints in advanced penile cancer truly reflect the response to immunotherapy? Will the process of combination therapy lead to a reprogramming of immune checkpoint expression? Are there differences in the expression of immune checkpoints and response to ICIs between metastatic and non-metastatic penile cancer? These uncertainties are the directions that all immunologists and urologists urgently want to resolve at present.

### 3.3 HPV infection status

HPV infection is associated with the occurrence of various malignant neoplasm (31). Its main carcinogenic mechanism is that high-risk HPV integrates its DNA into the host cell genome by infecting basal epithelial cells, leading to overexpression of viral oncogenic proteins (32). Early viral oncogenic proteins such as E6 and E7 in HPV are closely related to malignant phenotypes, such as cell cycle dysregulation, abnormal cell apoptosis, DNA damage repair stagnation, and inactivation of the P53 protein (33). Penile cancer can be divided into HPV+ disease and HPV- disease (34). In a meta-analysis of 4199 cases of penile cancer patients, HPV+ disease accounted for approximately half of the total cohort (35), mainly HPV types 16 and 18 (36). However, the prognostic significance of HPV infection status in penile cancer is still inconclusive, overall leaning towards better prognosis for HPV+ patients, possibly related to enhanced immune response to viral infection (37). There are some differences between HPV+ and HPV- cancers in their development and immune characteristics (38). HPV+ cancers typically have a higher invasion of immune cells, a higher burden of mutations, and an active immune response. This is because HPV infection activates the host’s immune system to attack the virus and infected cells. In contrast, HPV-cancers generally have a lower invasion of immune cells and a lower burden of mutations. Because HPV+ cancers have more significant immune activity, ICIs generally show better efficacy in such cancers. Additionally, the incidence of PD-L1 positivity in HPV- penile cancer is higher than in HPV+ penile cancer (39). These pieces of evidence collectively suggest that the lack of HPV infection may mediate cancer immune suppression or immune escape. In summary, while HPV-negative penile cancers may have higher PD-L1 expression, the efficacy of ICIs in HPV-positive penile cancer can be attributed to the presence of a more favorable immune microenvironment, including higher T cell infiltration and activation, and potentially different immune escape mechanisms. These factors collectively contribute to the observed clinical responses to ICIs in HPV-positive penile cancer, highlighting the complexity of immune interactions in cancer immunotherapy. Considering the continuous presence of HPV viral proteins throughout the entire lifecycle of cancer cells (40), developing therapeutic HPV vaccines targeting E6 and E7 oncogenic proteins is a novel and feasible approach. Therapeutic HPV vaccines deliver the oncogenic proteins to immune cells, enhancing the immune response of CD8+ cytotoxic T cells against HPV+ penile cancer (36), with promising prospects for application.

### 3.4 Tumor immune microenvironment

TIME refers to the complex milieu within and around tumors where various immune cells, stromal cells, and signaling molecules interact with cancer cells. Beyond immune cells, non-immune components like fibroblasts, blood vessels (angiogenesis), and extracellular matrix (ECM) proteins also influence the TME. They can provide structural support to tumors, modulate immune cell infiltration, and secrete cytokines that promote immune evasion (41). TIME is the foundation for understanding the potential drivers of disease and developing effective therapies. In penile cancer, understanding and leveraging the complex interactive characteristics of TIME will create conditions for the development of novel immune therapeutic strategies. TIME can either promote or inhibit cancer progression (41). A retrospective study involving 213 penile cancer patients found that a high level of infiltrating CD8+ cytotoxic T cells was associated with a low risk of lymph node metastasis (26). It is worth noting that the prognostic significance of FOXP3+ regulatory T cells in penile cancer is controversial (42, 43). Additionally, macrophages exhibit high plasticity in penile cancer. High levels of infiltrating CD163+ tumor-associated macrophages are associated with a high risk of lymph node metastasis (26), while high levels of infiltrating CD206+ or CD68+ tumor-associated macrophages are associated with a better prognosis (43, 44). Myeloid-derived suppressor cells (MDSCs) are also important components of the immunosuppressive microenvironment within cancers (45). MDSCs are a group of cells with phenotypic heterogeneity (CD11b+/CD33+) but lack differentiation ability, and they can secrete inhibitory factors to weaken the anti-cancer abilities of CD8+ T cells or NK cells (46). In other malignant cancers, MDSCs have been shown to be associated with patient chemoresistance, lack of response to ICIs, and poor prognosis (47, 48). In a mouse model of penile cancer, the proportion of MDSCs in cancer tissue is much higher than in non-cancer tissue (49). Therefore, it is necessary to continue researching the role of MDSCs in human penile cancer. In summary, the immune response in cancer is not simply a sum of its parts but a dynamic network of interactions among immune cells, non-immune cells, and tumor cells within the TIME. Understanding these interactions at a systems level is essential for developing more effective immunotherapies and personalized treatment strategies tailored to the unique immune landscape of each patient’s tumor. With the application of single-cell sequencing and spatial transcriptome sequencing, we will better understand the connections between various immune cells and cancer cells in the TIME of penile cancer, and drive the advancement of immune therapeutic strategies by manipulating these immune components.

## 4 Advances in clinical research on immunotherapy for penile cancer

### 4.1 The application of ICIs

NCT02721732 indicated that pembrolizumab (anti-PD-1) in combination with the TIP chemotherapy regimen and also underwent surgical consolidation provided relief for advanced penile cancer patients with high microsatellite instability (Disease-free survival: 38.7 months) (10). A retrospective study involving 17 advanced penile cancer patients found that the combination of toripalimab (anti-PD-1) with nivolumab plus TIP chemotherapy showed good anti-cancer feedback (2-year overall survival: 62.9%) (50). NCT03333616 reported that the combination of nivolumab (anti-PD-1) and ipilimumab (anti-CTLA-4) did not show a treatment response in 5 penile cancer patients (Objective response rate: 16%) (51), but the same combination regimen demonstrated a significant therapeutic response in another case of metastatic penile cancer progressing after chemotherapy (52). The use of nivolumab (anti-PD-1) in penile cancer with radiation resistant also showed some treatment response (53). NCT03686332 found that atezolizumab (anti-PD-L1) in combination with radiation therapy had some response in advanced penile cancer patients (Median follow-up: 29.1 months) (7). The application of ICIs in advanced penile cancer holds great promise. In the future, we look forward to more dual or triple combination strategies of ICIs with other therapies to explore the optimal treatment combinations in penile cancer.

### 4.2 The application of therapeutic HPV vaccines

Although there is currently no published research on the use of therapeutic HPV vaccines in penile cancer, therapeutic HPV vaccines have become an experimental treatment strategy for some HPV-related malignant cancers (54). For example, therapeutic HPV vaccines carrying the E7 antigen have generated anti-cancer immune responses in some advanced cervical cancer patients (55). In another clinical trial involving 1356 patients, the use of therapeutic HPV vaccines expressing the E2 antigen (negative regulation of E6 and E7) has shown good responses in patients with cervical intraepithelial neoplasia, genital warts, or anal intraepithelial neoplasia (56). With an increasing understanding of the structure of the HPV virus, more therapeutic HPV vaccines targeting different protein targets are being developed (11, 57, 58). Additionally, combination treatment strategies related to therapeutic HPV vaccines are also being explored (59). In summary, therapeutic HPV vaccines provide an opportunity for HPV-positive penile cancer patients to have a treatment option.

### 4.3 The application of adoptive cell therapy

ACT is a cancer immunotherapy that involves genetically modifying T cells to express chimeric antigen receptors (CAR-T) or T cell receptors (TCR-T) (60). The clinical trial NCT02858310 is targeting HPV E7 with TCR-T for the therapy of patients with metastatic HPV-related epithelial cancers (61). Notably, there were no patients with penile cancer included in the sample studied in this clinical trial (61). Encouragingly, half of the 8 patients with anti-PD-1 resistant tumors showed significant cancer regression (61). Similarly, NCT02280811 also found the potential of TCR-T to induce regression of epithelial cancers (62). It was worth noting that one patient with three lung metastases had complete regression of one metastatic lesion and partial regression of the other two lesions after TCR-T treatment, followed by surgical resection, and achieved a disease-free survival of 3 years (62). On the other hand, ACT using tumor-infiltrating lymphocytes (TILs) is a personalized cancer immunotherapy approach that utilizes TILs from the patient’s own tumor immune microenvironment (63). Studies have demonstrated the feasibility of expanding TILs from penile cancer patients, and their expansion phenotype is unrelated to chemotherapy (64). This supports the use of TILs-based ACT strategies in penile cancer patients who have progressed on chemotherapy. The use of TILs in penile cancer therapy is an area of emerging interest, but several limitations need to be considered: heterogeneity of penile cancer, limited clinical data, challenges in TILs extraction and expansion, immunosuppressive tumor microenvironment, patient selection and timing, long-term efficacy and safety and cost and accessibility. Addressing these limitations will be crucial for advancing TIL-based therapies in penile cancer, potentially offering new treatment options but requiring further research and development to establish their place in clinical care.

## 5 The pros and cons of cancer immunotherapy

Immunotherapy is a continuously evolving treatment for cancer. Despite its many advantages, due to its complexity and unpredictability, we still need to comprehensively consider the pros and cons of its application (65). Compared to chemotherapy and radiation therapy, immunotherapy has demonstrated a broader spectrum of treatment and a reduced incidence of treatment resistance in clinical efficacy (66). In recent years, it has become an important first or second-line treatment in penile cancer (6). Furthermore, combination therapy with immunotherapy and other treatments has shown to be more effective than using only one treatment approach (29). Immunotherapy aims to boost the patient’s immune system against cancer, resulting in minimal impact on normal cells and exhibiting strong specificity and fewer toxic side effects (21, 67). In addition, the durability of immunotherapy is also a key factor in helping patients achieve long-term anti-cancer benefits (68). Since the immune system can retain memory of attacking cancer cells and continue to attack them, patients may maintain lower levels of cancer cells for a long period of time after treatment. Although immunotherapy has significant advantages in cancer treatment, there are also some undeniable disadvantages that cannot be ignored (69). Immunotherapy is not applicable to all patients (70). Some patients may not derive benefits from immunotherapy or may experience significant side effects. These observations have been reported not only in penile cancer but also in other types of cancers. This highlights the significance of researching the molecular basis of response to cancer immunotherapy, which can greatly contribute to better identifying the patient populations that are likely to benefit from immunotherapy and to formulating appropriate indications for its use in treatment. Immunotherapy can potentially lead to serious side effects, such as immune-related toxic reactions (71). Therefore, it is necessary to assess the patient’s treatment tolerance and closely monitor them during the treatment process. Moreover, cancer immunotherapy is often associated with high costs, and it is important to consider its cost-effectiveness by taking into account factors such as treatment expenses, treatment efficacy, and expected patient survival time. In summary, while tumor immunotherapy offers numerous benefits, it is crucial to consider individual patient differences, treatment indications, treatment tolerance, and cost-effectiveness. By evaluating the advantages and disadvantages of its application, we can ensure that patients receive the most suitable and effective treatment options.

## 6 Conclusions

**Figure 1:**
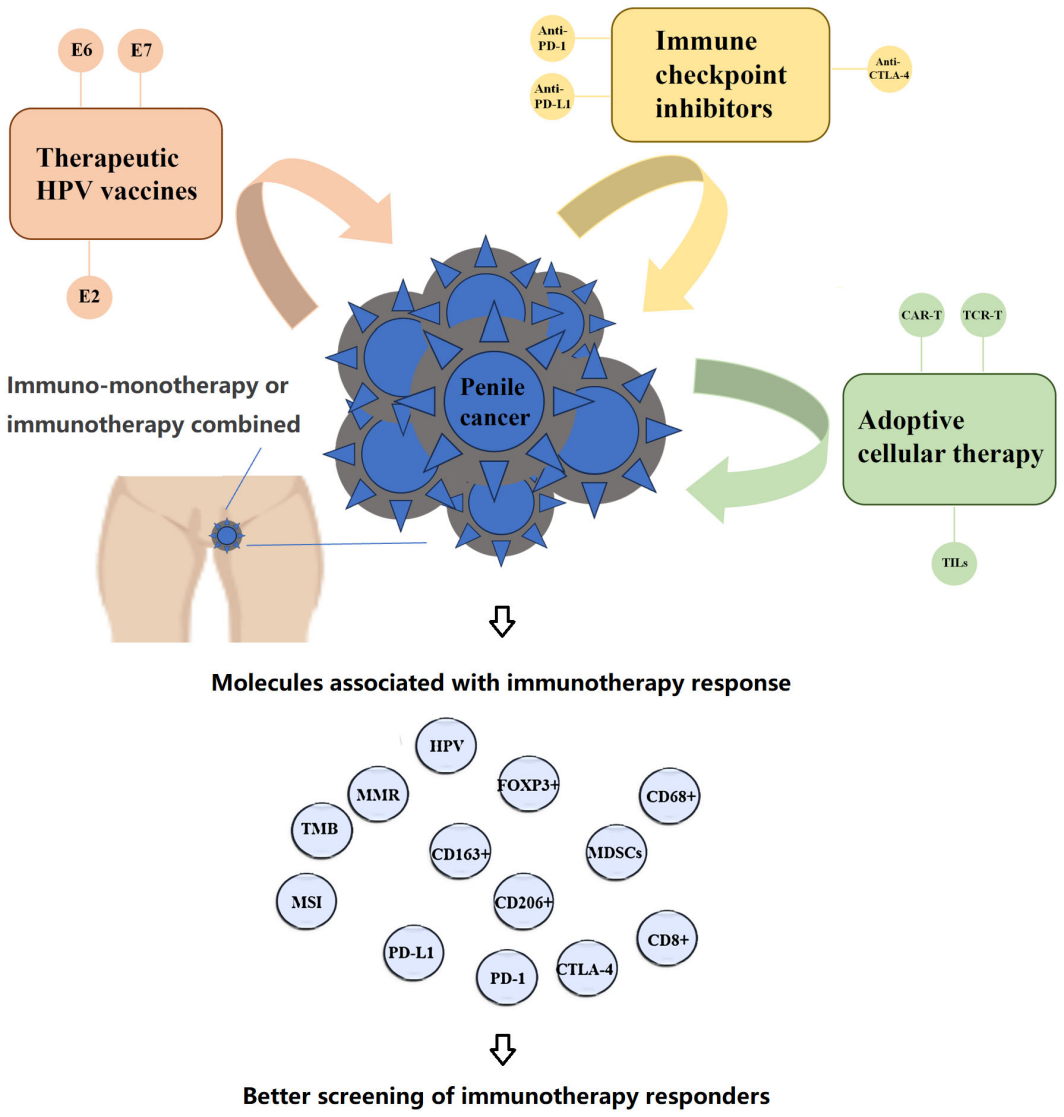
Molecular basis of response to immunotherapy for penile cancer.

This review focuses on immune-related novel adjuvant therapies for patients with advanced penile cancer, with a main emphasis on molecular biomarkers associated with immune therapy response (**Figure 1**). These biomarkers cover genomic features of penile cancer as well as gene expression characteristics of immune microenvironment cells. Our review provides a comprehensive overview, specifically exploring the value of these molecular biomarkers in predicting treatment efficacy and screening population response in immune therapy for patients with advanced penile cancer. We have also found that there is still a considerable gap in our understanding of the mechanisms underlying treatment response. Theoretically, there is an opportunity to use immunotherapy as neoadjuvant therapy for advanced penile cancer patients. However, current clinical trials of immunomonotherapy or combination immunotherapy strategies have not yielded definitive desirable results. As the immune microenvironment of penile cancer continues to be revealed, future research and development should focus on biomarkers that are relevant to the actual immunobiology of the tumor. These biomarkers can help us better evaluate patient responses to immunotherapy and predict treatment outcomes. By gaining a deeper understanding of the immune features of the tumor, we can more accurately determine which patients are likely to benefit from immunotherapy and how to better design individualized treatment plans.

## Author contributions

DX: Writing – original draft, Writing – review & editing. LC: Writing – original draft, Writing – review & editing. XZ: Supervision, Writing – original draft. HH: Conceptualization, Writing – review & editing. MM: Conceptualization, Writing – review & editing.
